# Integrating Wearable Temperature, Activity, Melanopic Light, and Lifelog Data to Assess Circadian–Behavioral Coupling in Free-Living Conditions

**DOI:** 10.3390/s26185703

**Published:** 2026-09-08

**Authors:** Sora Matsumoto, Yuki Hashimoto, Yoshifumi Nishida

**Affiliations:** 1 Department of Mechanical Engineering, School of Engineering, Institute of Science Tokyo, Tokyo 1520033, Japannishida.y.af@m.titech.ac.jp (Y.N.); 2 Institute of Biomedical Engineering, Smart Hospital Healthcare, Institute of Science Tokyo, Tokyo 1520033, Japan

**Keywords:** wearable sensors, circadian rhythm, daily behavioral rhythm, core body temperature, actigraphy, melanopic EDI, multi-sensor data fusion, circadian–behavioral coupling

## Abstract

Circadian–behavioral misalignment, defined as a mismatch between internal circadian timing and daily sleep activity behavior, is associated with sleep quality, daytime function, cognitive performance, and long-term health risks. However, practical approaches for integrating circadian-phase-related physiological markers with behavioral and environmental sensing under free-living conditions remain limited. This study presents a multimodal wearable and lifelog monitoring workflow that combines a heat-flux-based wearable temperature-estimation sensor, a wrist-worn actigraph, melanopic equivalent daylight illuminance (EDI) measurement, and web-based lifelog entries. Ten healthy young adults were monitored for approximately one week in daily life. The midpoint between the descending and ascending mid-level crossings of the nocturnal temperature rhythm was extracted as a circadian-phase-related temperature rhythm marker. In a practical comparison with gastrointestinal temperature measured using an ingestible capsule, the wearable-derived rhythm marker showed a mean absolute difference of 27.1 ± 16.6 min. The temporal difference between the two markers was defined as the phase angle, and coupling stability was evaluated using phase angle variability and the correlation between the markers. After excluding one participant who traveled overseas, clustering and principal component analyses were used to visualize exploratory participant-level differences in circadian–behavioral coupling. Integrated analysis with melanopic EDI, activity, and lifelog-derived features suggested that light exposure, activity timing, and behavioral timing may provide contextual information for interpreting coupling patterns in this small cohort. These findings support the feasibility of a sensing system application that integrates physiological, behavioral, environmental, and lifelog data for the exploratory assessment of circadian–behavioral coupling under free-living conditions, rather than the development of new sensor hardware.

## 1. Introduction

**Figure 1:**
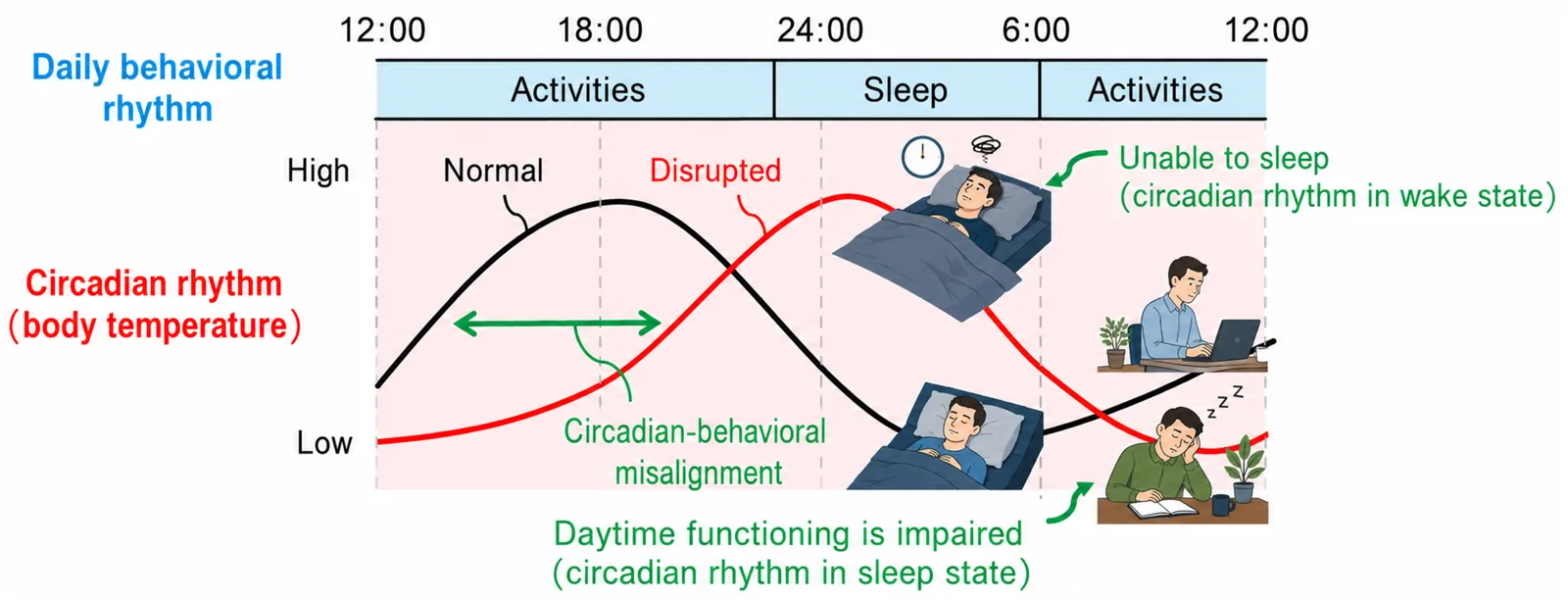
Conceptual illustration of circadian–behavioral misalignment. The black curve represents a normally aligned circadian-phase-related physiological rhythm, whereas the red curve represents a shifted or disrupted circadian rhythm relative to the daily behavioral schedule. The blue blocks indicate daily behavioral timing, including activity and sleep periods. The green arrows and annotations illustrate the temporal mismatch between circadian and behavioral rhythms and examples of the associated functional consequences. This figure was created by the authors as a conceptual schematic based on previous descriptions of circadian misalignment and the relationship between sleep–wake timing, circadian physiology, and body temperature rhythms [8,9,10].

Human physiological functions are temporally regulated by circadian rhythms with an approximate 24 h periodicity [1,2]. Circadian rhythms are involved in sleep–wake behavior, core body temperature, hormone secretion, metabolism, cognitive function, and other physiological processes, and they are maintained through entrainment between the endogenous biological clock and the external environment [2,3,4]. In modern society, shift work, socially constrained schedules, artificial light exposure at night, electronic media use before bedtime, and irregular eating and activity schedules can generate mismatches between endogenous circadian rhythms and daily behavioral rhythms [2,5,6,7]. Figure 1 provides a conceptual schematic of circadian–behavioral misalignment, based on previous descriptions of misalignment between sleep–wake behavior and circadian physiology, including body temperature rhythms [8,9,10]. Such misalignment has been associated with poor sleep quality, daytime sleepiness, impaired attention and cognitive performance, mood disturbance, and long-term metabolic and cardiovascular health risks [2,5,11,12].

Rhythm assessment under free-living conditions has traditionally relied mainly on actigraphy for the long-term monitoring of sleep–wake patterns and physical activity [13,14,15]. Actigraphy is noninvasive and well suited to the long-term monitoring of sleep and activity rhythms in daily life. However, actigraphy mainly reflects behavioral rhythms and does not directly provide the endogenous circadian phase. Therefore, sleep timing may appear regular while its phase relationship with the internal circadian rhythm is unstable, or conversely, variable sleep timing may still maintain a stable relation to circadian phase. A method that simultaneously measures circadian-phase-related physiological signals and daily behavioral rhythms is therefore needed to visualize and quantify their coupling under free-living conditions.

Core body temperature has been widely used as a physiological indicator of circadian phase [3,16,17]. It typically shows a clear daily rhythm, with higher values in the evening and before bedtime, a decline during sleep, and a minimum in the early morning or around wake time. The phase and amplitude of this rhythm are important markers for circadian assessment [3,17,18]. The strict assessment of core body temperature has used measurement sites close to central circulation, such as esophageal, pulmonary artery, rectal, or bladder temperature. These approaches are limited for repeated long-term daily life monitoring because of invasiveness, measurement burden, and restrictions on measurement duration [17,19,20]. Ingestible temperature capsules are practical for free-living studies, but their values are affected by gastrointestinal location, transit time, food and fluid intake, local intestinal conditions, and temporal response; therefore, they should not be treated as an absolute gold-standard reference for true core body temperature [19,20,21].

Recent advances in heat-flux-based patch sensors and wearable core-temperature-estimation technologies have enabled the noninvasive monitoring of core-temperature-related rhythms under free-living conditions [20]. However, previous studies have primarily evaluated sensor-level agreement during heat stress, exercise, or controlled laboratory protocols, and estimation performance may be affected by activity intensity, environmental conditions, and sensor attachment state [22,23,24,25,26,27,28,29]. In parallel, wearable monitoring has expanded beyond conventional accelerometry to include multimodal physiological, behavioral, and environmental sensing, such as wrist-worn actigraphy, optical and photonic sensors for cardiovascular and respiratory monitoring, wearable light sensors, and patch-type temperature or heat-flux sensors [29,30]. Nevertheless, these sensing modalities are often examined separately, and practical frameworks for synchronizing and integrating physiological, behavioral, environmental, and self-reported data remain limited, particularly for assessing circadian–behavioral relationships in daily life.

The aim of this study was to develop and evaluate a wearable multi-sensor and lifelog data-integration workflow for assessing circadian–behavioral coupling under free-living conditions. The workflow integrates a heat-flux-based wearable temperature-estimation sensor, a wrist-worn actigraph, melanopic EDI measurement, and web-based lifelog entries on a common time axis. The midpoint between the descending and ascending mid-level crossings of the nocturnal temperature rhythm was used as a circadian-phase-related temperature rhythm marker, and midsleep time was used as a behavioral rhythm marker. Their temporal difference was defined as the phase angle, and coupling stability was evaluated using day-to-day phase-angle variability and the correlation between the two rhythm markers.

The contributions of this study are threefold. First, we implemented a common-time-axis workflow for synchronizing wearable and lifelog data collected in daily life. Second, we evaluated the practical agreement between wearable and gastrointestinal-temperature-derived rhythm markers, treating the ingestible capsule as a practical comparator rather than an absolute gold standard. Third, we integrated physiological, behavioral, environmental, and lifelog-derived features to contextualize individual differences in circadian–behavioral coupling. Collectively, the contribution lies in sensor–system integration, feature extraction, and multi-sensor data fusion, rather than in the development of new sensor hardware.

## 2. Wearable System and Rhythm Definitions

### 2.1. Wearable System

Wearable systems for physical well-being monitoring commonly combine physiological, behavioral, and environmental sensors. Physiological signals can include body temperature [20], cardiac activity [30], oxygen saturation [31], sweat rate and electrolyte concentration [32], and electromyographic activity [33], whereas accelerometry and actigraphy are widely used to assess movement and sleep–wake behavior [13]. Light and ambient-temperature sensors provide additional information on environmental exposure. Recent advances also include flexible and printed sensors, such as aerosol-jet-printed electromyographic electrodes intended to improve conformability and signal stability [34]. Within this broad field, the present study focuses on the synchronized integration of temperature-related sensing, actigraphy, melanopic light exposure, and lifelog data for circadian–behavioral rhythm assessment.

**Figure 2:**
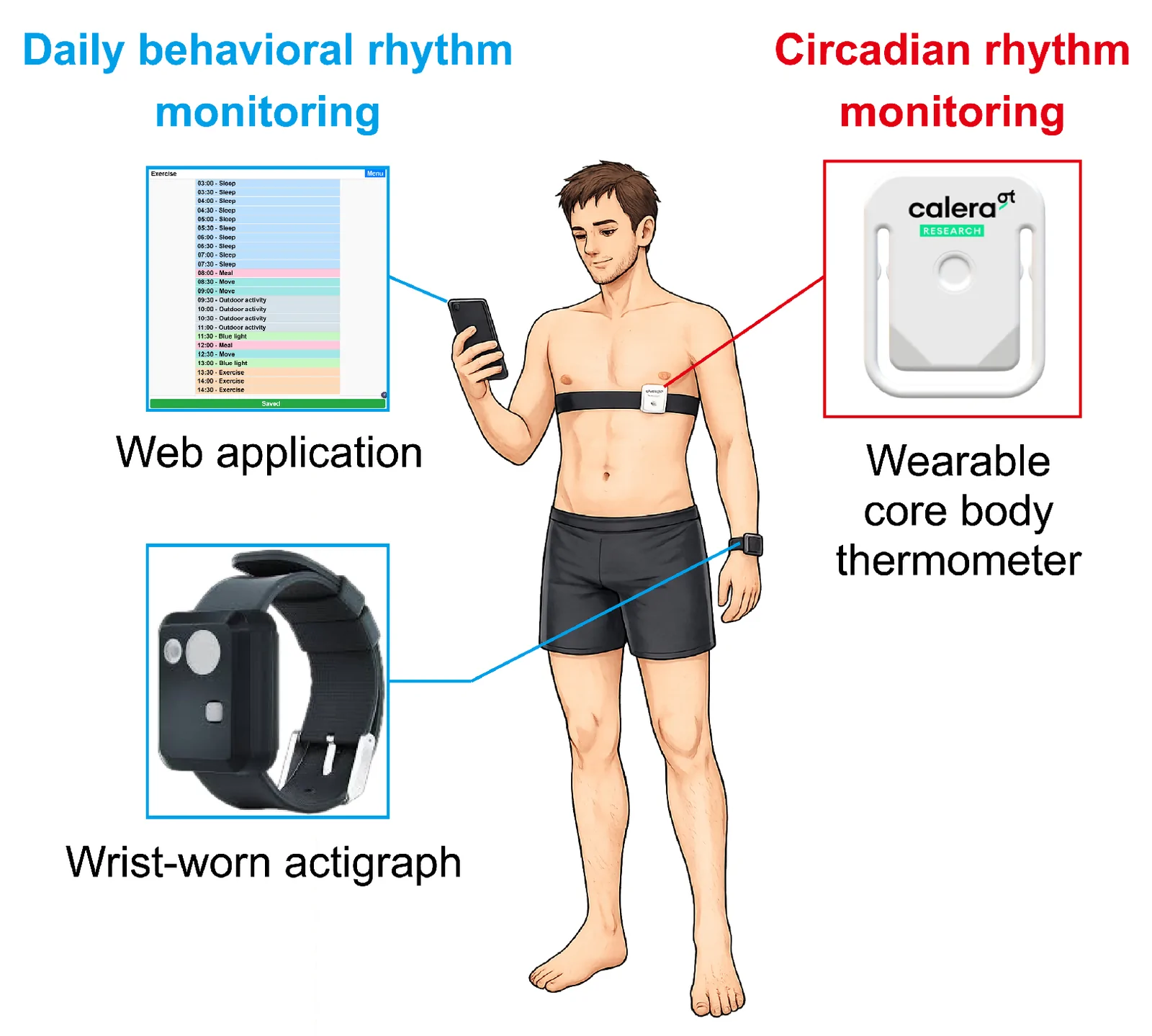
Overview of the multimodal wearable sensing system. The system integrates a heat-flux-based wearable temperature-estimation sensor, a wrist-worn actigraph that records activity and melanopic EDI, and a web application for 30 min lifelog entries. The e-Celsius capsule was used as a practical gastrointestinal temperature comparator under free-living conditions, not as a strict gold-standard reference for core body temperature.

The wearable multi-sensor monitoring system and data-integration workflow are shown in Figure 2. The system consisted of a heat-flux-based wearable core-temperature-estimation sensor (CALERA Research, greenTEG), a wrist-worn actigraph (ActLumus, Condor Instruments), and a web application for lifelog recording. The temperature-estimation sensor was worn on the lateral chest at the manufacturer-recommended position using the supplied strap, and derived a core-temperature-related estimate from local skin temperature and heat flux.

The wearable output served as a noninvasive temperature rhythm signal for marker extraction rather than as an absolute measure of true core body temperature. Estimated values may be affected by attachment site, strap tightness, skin contact pressure, sweating, clothing, bedding, and the local thermal microenvironment.

The wrist-worn actigraph recorded physical activity, melanopic equivalent daylight illuminance (melanopic EDI), and ambient temperature. To reduce participant burden, the web application allowed participants to record sleep, meals, bathing, and other daily behaviors in 30-min intervals using their own mobile devices. These entries were used as contextual behavioral information rather than for minute-level event timing.

### 2.2. Extraction of Circadian Rhythm Features

**Figure 3:**
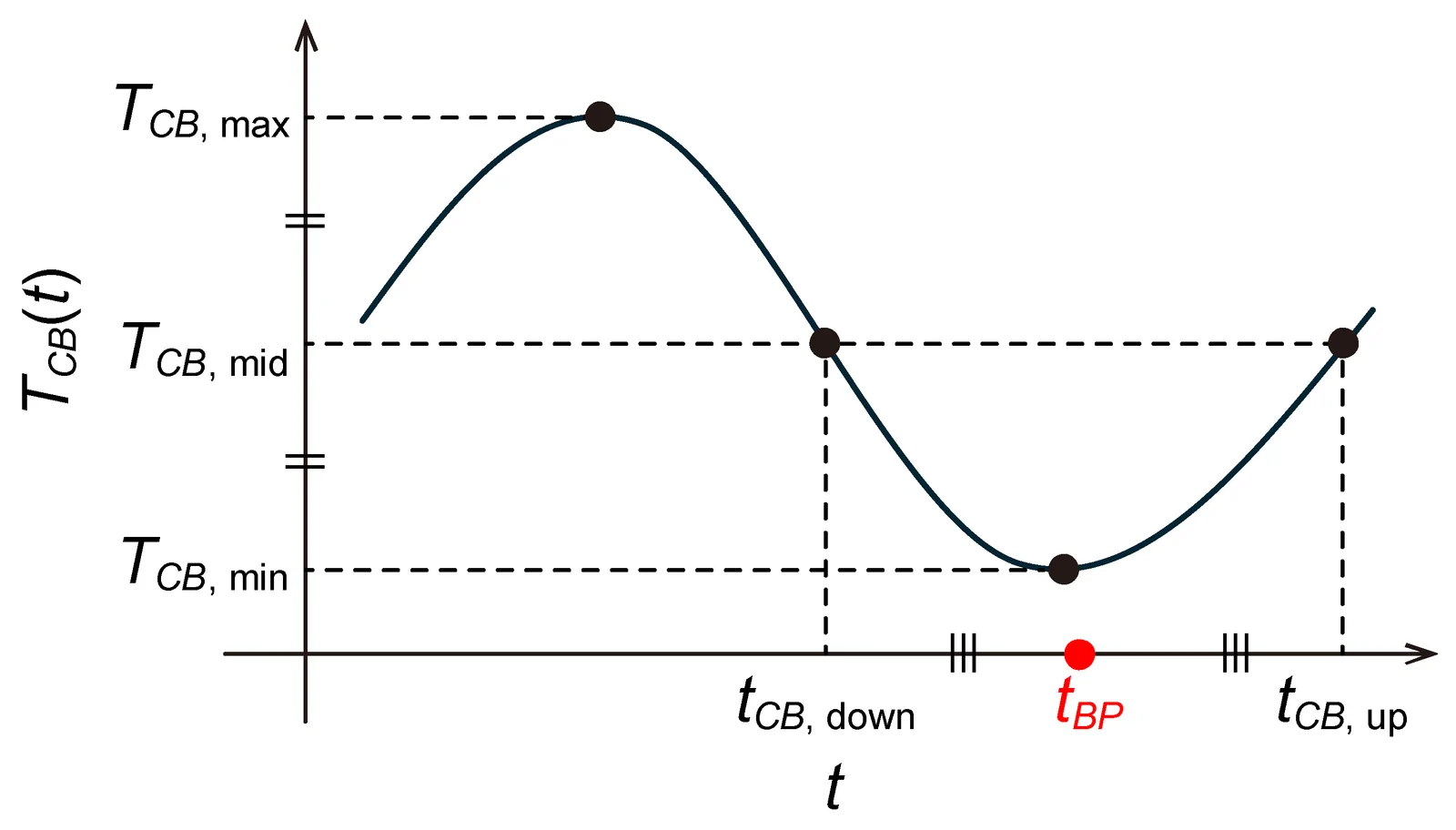
Definition of the circadian rhythm marker *t*_BP_. The temperature rhythm marker *t*_BP_ was calculated as the temporal midpoint between the descending and ascending mid-level crossings around the nocturnal temperature trough. The red marker indicates *t*_BP_. The horizontal dashed lines indicate *T*_CB,max_, *T*_CB,mid_, and *T*_CB,min_, and the vertical dashed lines indicate the descending and ascending mid-level crossing times, *t*_CB,down_ and *t*_CB,up_, respectively.

The core-body-temperature-related estimate obtained from the wearable temperature-estimation sensor is denoted as *T_CB_*(*t*) and was used to derive the daily temperature rhythm. As illustrated in Figure 3, *t_BP_* was defined as the temporal midpoint between the descending and ascending mid-level crossings around the nocturnal temperature trough [35,36]. For each preprocessed daily segment, the maximum and minimum values of the temperature rhythm, *T_CB,max_* and *T_CB,min_*, were identified. The mid-level temperature, *T_CB,mid_*, was calculated as their arithmetic midpoint:

(1) $$T_{CB,mid}=\frac{T_{CB,max}+T_{CB,min}}{2}$$

The descending and ascending crossing times were then identified on either side of the nocturnal trough. The descending crossing time, *t_CB,down_*, was defined as the time at which the smoothed temperature curve reached, or came closest to, *T_CB,mid_* during the descending portion of the rhythm. Similarly, the ascending crossing time, *t_CB,up_*, was defined during the ascending portion;

(2) $$t_{CB,down}=\underset{t<t_{\mathrm{CB},\min}}{\mathrm{argmin}}\left\{\left|t-t_{CB,\;min}\right||T_{CB}\left(t\right)=T_{CB,mid}\right\}$$

(3) $$t_{CB,up}=\underset{t>t_{\mathrm{CB},\min}}{\mathrm{argmin}}\left\{\left|t-t_{CB,\;min}\right||T_{CB}\left(t\right)=T_{CB,mid}\right\}$$

The temperature rhythm marker *t_BP_* was calculated as the temporal midpoint between these two crossing times;

(4) $$t_{BP}=\frac{t_{CB,down}+t_{CB,up}}{2}$$

This definition characterizes the timing of the nocturnal temperature rhythm without assuming a perfectly periodic waveform. Accordingly, *t_BP_* was interpreted as a practical circadian-phase-related marker derived from the wearable temperature rhythm, rather than as a direct estimate of the biological core body temperature minimum.

### 2.3. Extraction of Daily Behavioral Rhythm Features

Midsleep time (*t_sleep,mid_*) was used as the behavioral timing marker corresponding to the circadian-phase-related temperature rhythm marker *t_BP_* [37,38]. It was calculated as the midpoint between sleep onset time, *t_sleep,onset_*, and sleep offset time, *t_sleep,offset_*;

(5) $$t_{sleep,mid}=\frac{t_{sleep,\;onset}+t_{sleep,\;offset}}{2}$$

Sleep onset and offset were estimated from wrist actigraphy using the Cole–Kripke algorithm [39]. Because *t_sleep,mid_* is also a clock-time variable, its day-to-day variability was analyzed using circular statistics to account for wrap-around at the 24 h boundary, as described in Section 3.3.

### 2.4. Operational Definition of Circadian-Behavioral Coupling

In this study, circadian–behavioral coupling was operationally defined as the temporal relationship between the circadian-phase-related temperature rhythm marker *t_BP_* and the behavioral timing marker *t_sleep,mid_*. For each day, the phase angle was calculated as the within-day temporal difference between the two markers after aligning them to the same daily cycle;

(6) $$phase\;angle=t_{BP}-t_{sleep,mid}$$

At the participant level, coupling stability was characterized using the standard deviation of the daily phase angle and the correlation coefficient between *t_BP_* and *t_sleep,mid_*. Lower phase-angle variability indicated a more stable temporal relationship between the temperature and behavioral rhythms, whereas the correlation coefficient indicated the extent to which the two markers shifted in the same direction across days. The statistical treatment of clock time and phase-angle variability is described in Section 3.3.

## 3. Experimental Methods

### 3.1. Participants

Ten healthy young adults voluntarily participated in this study (eight men and two women; age, 23.1 ± 1.2 years). All participants received a detailed explanation of the experimental procedures and potential risks and provided written informed consent before participation. All experimental procedures and protocols conformed to the Declaration of Helsinki and were approved by the Ethics Review Committee of Institute of Science Tokyo (approval number: 2025160). Measurements were conducted from October 2025 to March 2026.

This small, demographically homogeneous cohort was selected for a feasibility evaluation of the proposed multimodal monitoring workflow, and was not intended to establish population-level reference values or generalizable participant phenotypes.

### 3.2. Experimental Protocols

Temperature and behavioral rhythms were monitored for approximately one week under free-living conditions, except during bathing. The wearable temperature-estimation sensor and actigraph recorded the variables described in Section 2 at 1 min intervals. Participants recorded sleep, meals, bathing, and other daily behaviors using the web-based lifelog application at 30 min intervals.

As a practical comparator, participants swallowed an ingestible thermometer (e-Celsius Performance Pill, BodyCAP) with water before the monitoring period, and gastrointestinal temperature was recorded at 1 min intervals until capsule excretion [17]. These measurements were used to compare the wearable- and capsule-derived temperature rhythm markers and were not treated as an absolute reference standard for true core body temperature or as a direct validation of the absolute accuracy of the wearable sensor.

### 3.3. Data Analysis

Before feature extraction, timestamps from the wearable temperature-estimation sensor, ingestible capsule, actigraph, and lifelog were synchronized to a common time axis. Bathing periods, obvious nonwear periods, missing device data, and blank data sections were excluded.

Temperature data from the wearable sensor and ingestible capsule were resampled as 10 min averages to reduce noise, and were smoothed using a centered moving average spanning 30 min before to 30 min after each time point. These preprocessing parameters were selected for rhythm-level feature extraction rather than minute-by-minute sensor agreement, and were not optimized using the present dataset. Because no sensitivity analysis was performed, the selected configuration should not be considered optimal; its robustness to alternative smoothing windows should be evaluated in future studies.

For the ingestible capsule, only data obtained more than 6 h after ingestion were analyzed to reduce acute effects associated with water and food intake and early gastrointestinal transit [18,19]. Although this cutoff improved the practical comparability of the wearable- and capsule-derived rhythm markers, it did not eliminate variation related to capsule location, gastrointestinal transit, food or fluid intake, or local intestinal conditions.

The temperature rhythm marker *t_BP_* was calculated from the preprocessed wearable temperature data using (1)–(4). Midsleep time, *t_sleep,mid_*, was calculated from sleep periods identified by the Cole–Kripke algorithm using (5) [39]. Agreement was evaluated as the temporal difference between wearable-derived and capsule-derived *t_BP_* for daily segments in which both temperature data streams were available after preprocessing and after exclusion of the first 6 h following capsule ingestion.

The analyses focused primarily on daily and participant-level features. Minute-level Bland–Altman analyses treating repeated observations as independent and detailed error modeling during behavioral or physiological state transitions were not performed. Accordingly, the results describe a practical agreement between temperature rhythm markers rather than time-resolved sensor error mechanisms.

Sub 1 was excluded from the main clustering and PCA analyses because the monitoring period included overseas travel and was considered a special case reflecting re-entrainment after time-zone exposure. For the remaining participants, daily *t_sleep,mid_*, *t_BP_*, and the phase angle (*t_BP_* − *t_sleep,mid_*) were calculated. Because *t_BP_* and *t_sleep,mid_* are clock time variables that can wrap around the 24 h boundary, their day-to-day variability was quantified using circular standard deviations. In contrast, the phase angle was calculated after aligning both markers to the same daily cycle, and was therefore summarized using the standard deviation in hours. Circular statistics for the phase angle may be useful in future studies involving phase relationships that span the 24 h boundary.

Hierarchical clustering using Ward’s method [40] was performed after standardizing the following circadian–behavioral coupling features: circular standard deviation of *t_sleep,mid_*, circular standard deviation of *t_BP_*, standard deviation of the phase angle, mean absolute day-to-day change in *t_sleep,mid_*, mean absolute day-to-day change in *t_BP_*, and the correlation coefficient between *t_sleep,mid_* and *t_BP_*. The analysis was used to visualize relative participant-level differences in coupling patterns. Because the clustering was unsupervised and no external ground-truth labels were available, the resulting groups were interpreted as exploratory and hypothesis-generating rather than as confirmed participant categories.

An integrated principal component analysis (PCA) [41] was conducted to explore associations between circadian–behavioral coupling and environmental or behavioral features. The additional variables included pre-sleep melanopic EDI, daily duration of exposure to melanopic EDI ≥ 250 lx, the percentage of sleep time under melanopic EDI ≤ 10 lx, mean daily total proportional integration mode (PIM), day-to-day variability in the PIM-weighted activity center time, variability in the timing of major daily behaviors, and the duration of blue-light-related behavior between 18:00 and 24:00. These variables were combined with the coupling features, standardized, and analyzed using PCA. Given the small sample size, PCA was used only for the exploratory visualization of participant-level multimodal feature space; loadings were interpreted descriptively rather than for confirmatory inference, phenotype validation, or causal attribution.

## 4. Results

### 4.1. Agreement of Circadian Estimation

Figure S1 presents the temperatures recorded by the wearable temperature-estimation sensor and ingestible capsule for each participant, together with sleep periods detected by the wrist-worn actigraph. The ingestible capsule recording duration was 24.8 ± 12.6 h (mean ± SD), reflecting individual differences in the time to capsule excretion [21]. The eligible comparison window extended from 6 h after ingestion until the capsule recording ceased.

Table 1 contains 10 paired daily *t_BP_* estimates from eight participants, including only daily segments for which both wearable sensor and ingestible capsule data were available after preprocessing. Thus, the table represents a short-term comparison of temperature rhythm markers, and does not include the full approximately one-week monitoring period or assess minute-by-minute sensor accuracy.

The mean absolute difference between wearable- and capsule-derived *t_BP_* was 27.1 ± 16.6 min. Because the observed difference may reflect not only the wearable sensor’s estimation error but also gastrointestinal measurement location, temporal response, local thermal conditions, and sensor attachment state, it should be interpreted as a practical agreement between the two derived rhythm markers rather than as the absolute error relative to true core body temperature.

### 4.2. Visualization of Rhythm Interaction

**Figure 4:**
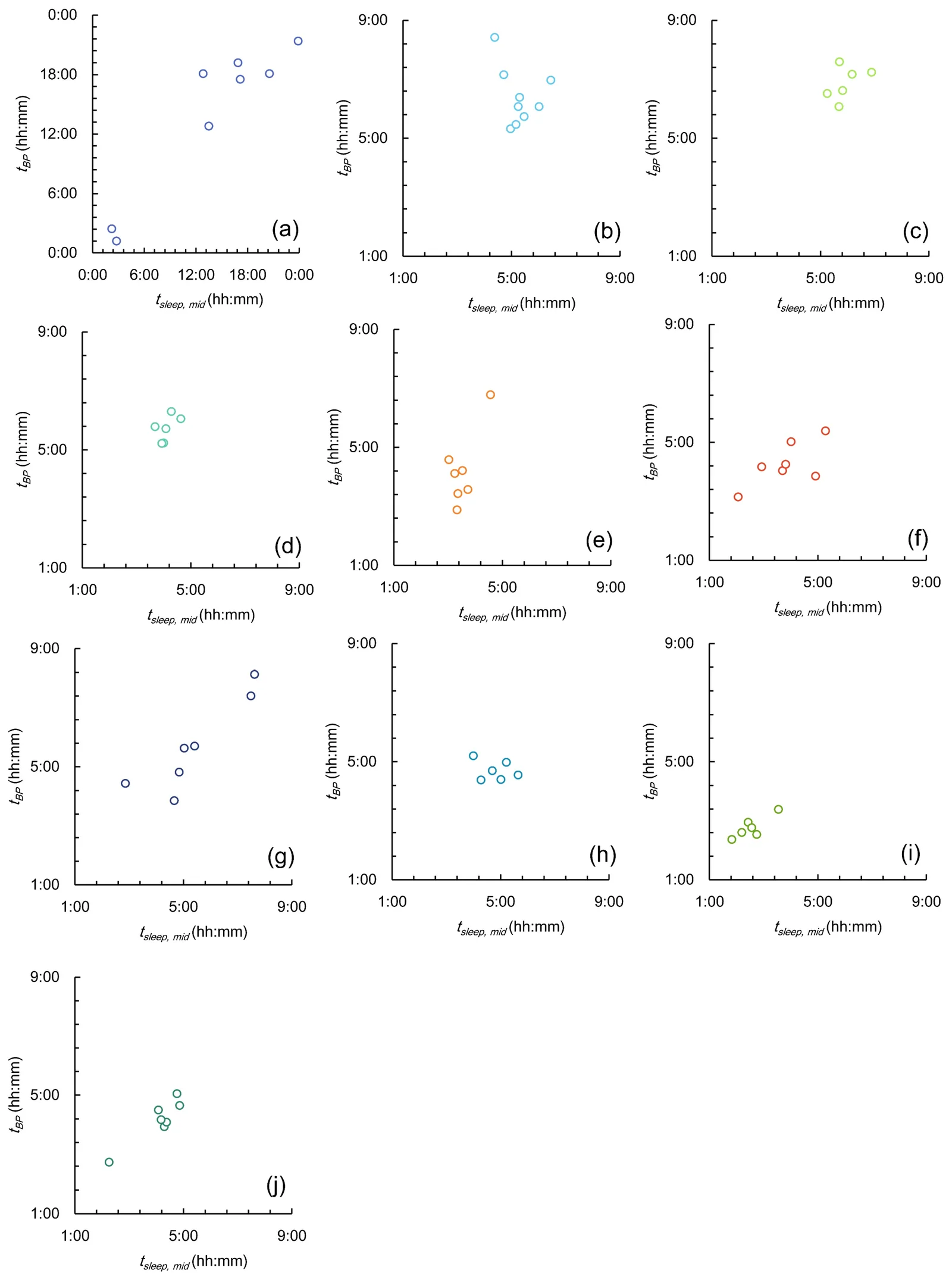
Day-by-day correspondence between the wearable-derived temperature rhythm marker *t_BP_* and actigraphy-derived midsleep time *t_sleep,mid_* for each participant. Each point represents one valid daily pair obtained during the approximate one-week monitoring period. The e-Celsius data were not used in this figure. (**a**) Sub 1, (**b**) Sub 2, (**c**) Sub 3, (**d**) Sub 4, (**e**) Sub 5, (**f**) Sub 6, (**g**) Sub 7, (**h**) Sub 8, (**i**) Sub 9, and (**j**) Sub 10.

Figure 4 shows all valid daily pairs of wearable-derived *t_BP_* and actigraphy-derived *t_sleep,mid_* obtained during the approximate one week monitoring period, separately for each participant. Unlike Table 1, Figure 4 does not use e-Celsius data. Figure 4a–j correspond to Sub 1–Sub 10, respectively. In most participants, both markers were concentrated in the morning hours, whereas Sub 1, who traveled overseas during monitoring, showed greater dispersion.

**Figure 5:**
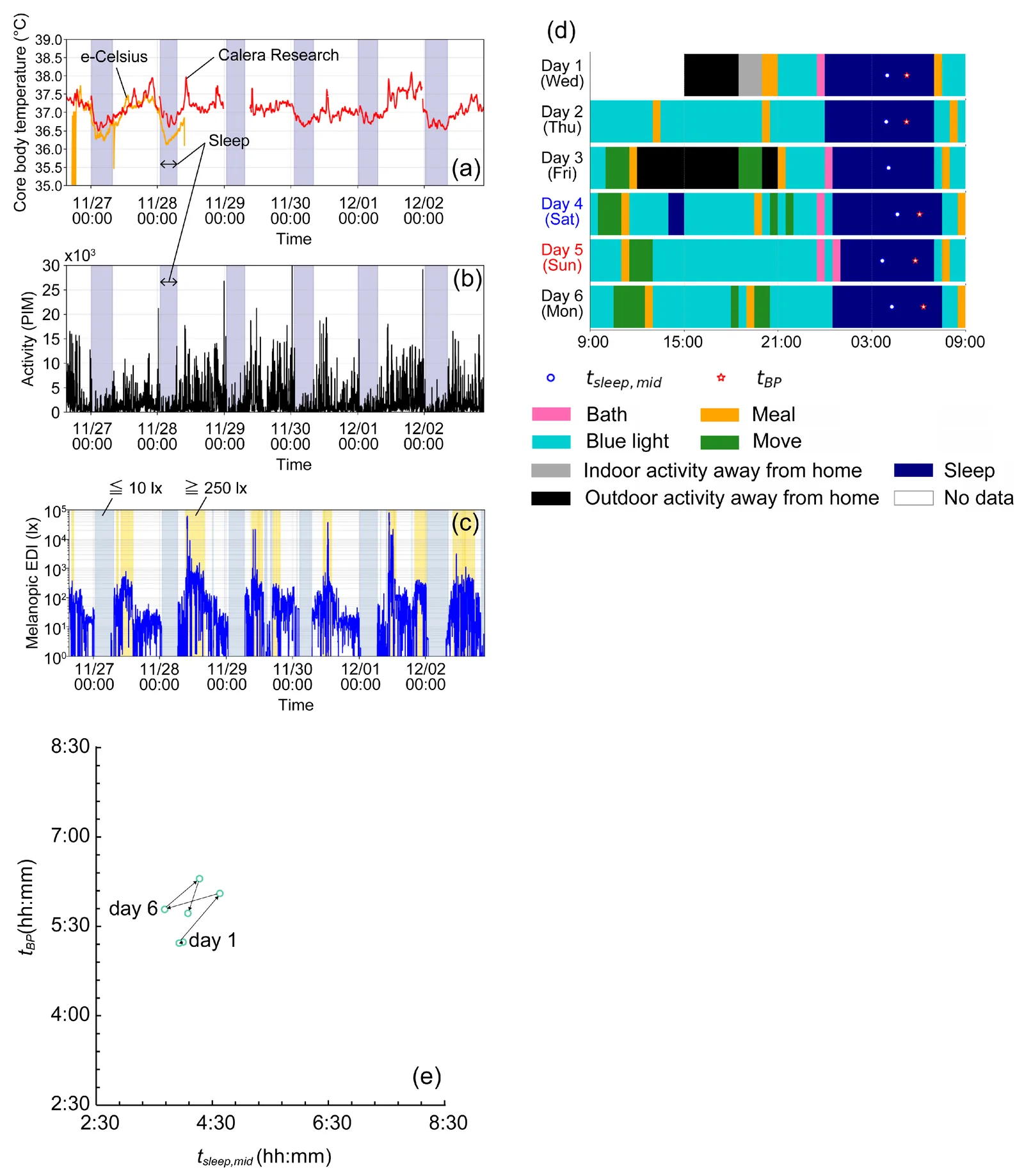
Representative time-series profiles and rhythm-marker relationships for Sub 4. The figure includes wearable-derived core-body-temperature-related estimates using CALERA Research and gastrointestinal temperature recorded using the e-Celsius capsule (**a**), physical activity expressed as PIM (**b**), melanopic EDI (**c**), lifelog-derived behavioral categories with daily *t_sleep,mid_* and *t_BP_* markers (**d**), and the day-by-day relationship between *t_BP_* and *t_sleep,mid_* (**e**). Shaded regions in (**a**,**b**) indicate sleep periods. In (**c**), yellow and blue shaded regions indicate periods with melanopic EDI ≤ 10 lx and melanopic EDI ≥ 250 lx, respectively.

**Figure 6:**
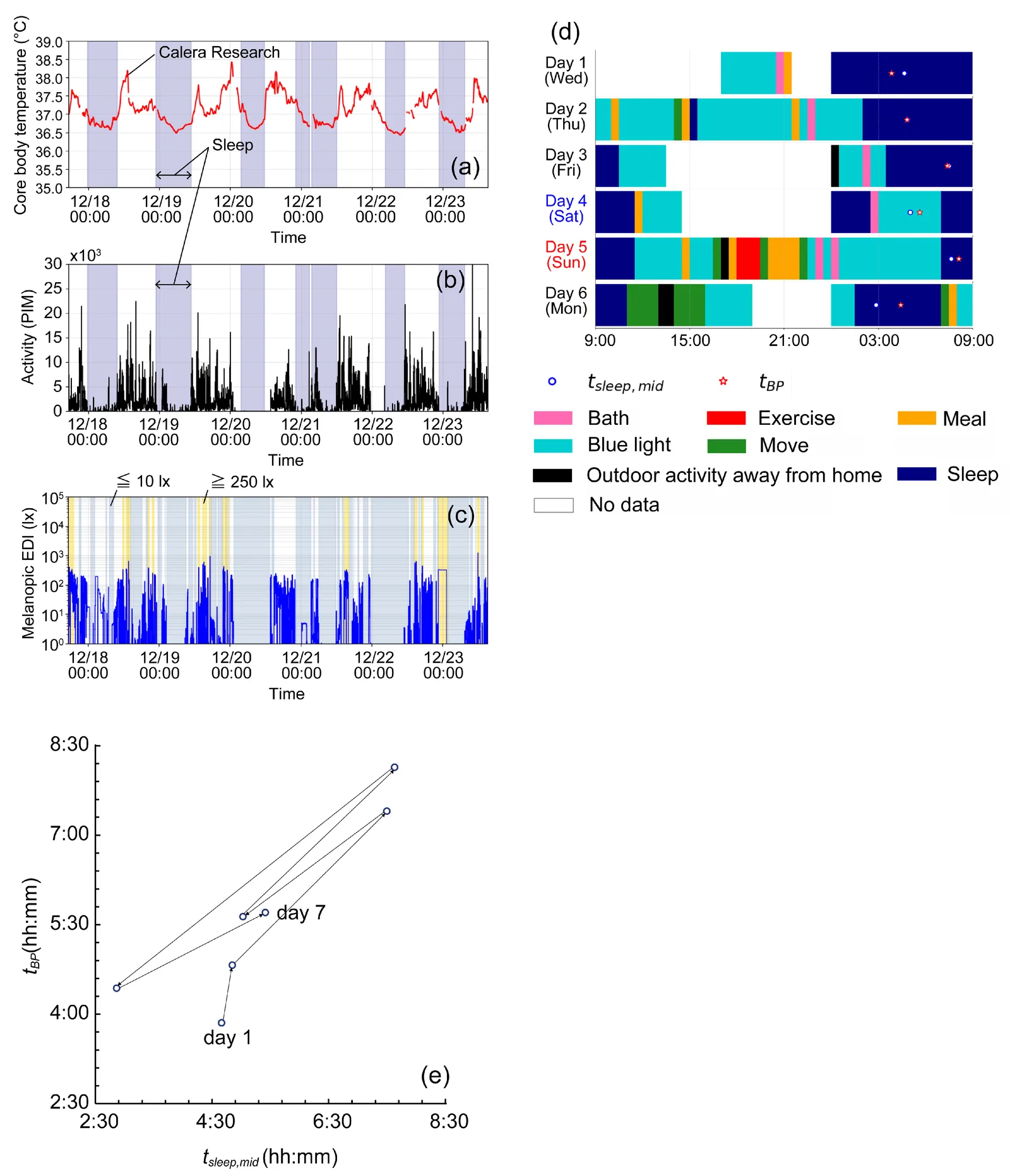
Representative time-series profiles and rhythm-marker relationships for Sub 7. The figure includes wearable-derived core-body-temperature-related estimates using CALERA Research and gastrointestinal temperature recorded using the e-Celsius capsule (**a**), physical activity expressed as PIM (**b**), melanopic EDI (**c**), lifelog-derived behavioral categories with daily *t_sleep,mid_* and *t_BP_* markers (**d**), and the day-by-day relationship between *t_BP_* and *t_sleep,mid_* (**e**). Shaded regions in (**a**,**b**) indicate sleep periods. In (**c**), yellow and blue shaded regions indicate periods with melanopic EDI ≤ 10 lx and melanopic EDI ≥ 250 lx, respectively.

**Figure 7:**
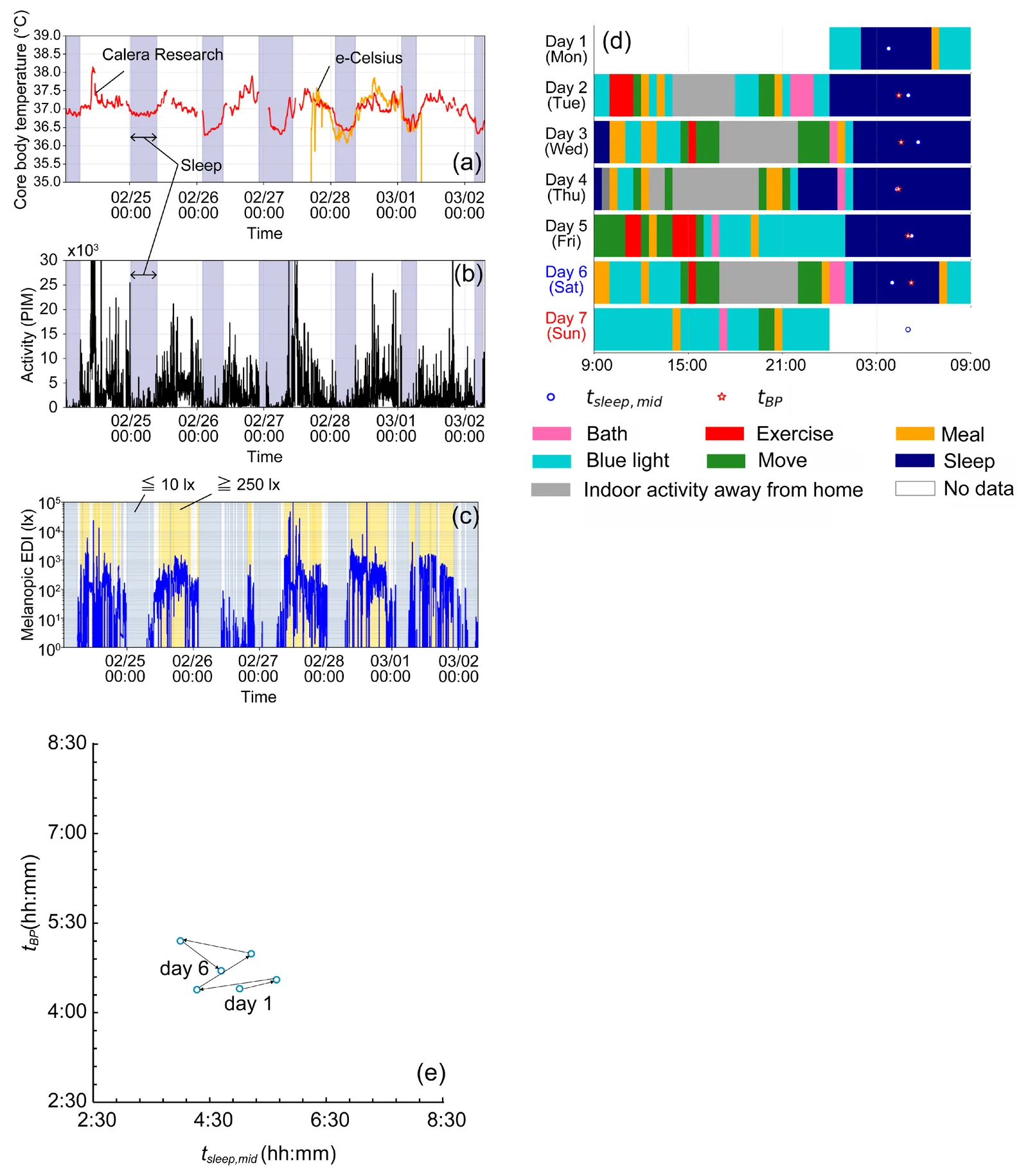
Representative time-series profiles and rhythm-marker relationships for Sub 8. The figure includes wearable-derived core-body-temperature-related estimates using CALERA Research and gastrointestinal temperature recorded using the e-Celsius capsule (**a**), physical activity expressed as PIM (**b**), melanopic EDI (**c**), lifelog-derived behavioral categories with daily *t_sleep,mid_* and *t_BP_* markers (**d**), and the day-by-day relationship between *t_BP_* and *t_sleep,mid_* (**e**). Shaded regions in (**a**,**b**) indicate sleep periods. In (**c**), yellow and blue shaded regions indicate periods with melanopic EDI ≤ 10 lx and melanopic EDI ≥ 250 lx, respectively.

Figure 5, Figure 6 and Figure 7 show representative time-series data for Sub 4, Sub 7, and Sub 8, including core-temperature-related estimates, activity, melanopic EDI, lifelog entries, and the daily relationship between *t_BP_* and *t_sleep,mid_*. Sub 4 (Figure 5) showed low variability in both markers, Sub 7 (Figure 6) showed large but coordinated day-to-day shifts, and Sub 8 (Figure 7) showed little tendency for the two markers to shift in the same direction. Phase-angle variability and the correlation between *t_BP_* and *t_sleep,mid_* are summarized in Table 2 and Table S1. Complete time-series data for all participants are provided in Figures S1 and S2.

### 4.3. Quantification of Coupling Stability

Representative circadian–behavioral coupling features and cluster assignments for the nine participants excluding Sub 1 are summarized in Table 2. The complete feature set, including day-to-day changes in *t_BP_* and *t_sleep,mid_*, is provided in Table S1. Sub 7 showed relatively large circular standard deviations of both markers, but maintained a high positive correlation between them, indicating large but coordinated day-to-day shifts. In contrast, Sub 2 and Sub 8 showed negative correlations, indicating that the two markers tended not to shift in the same direction. Given the small and homogeneous sample, all clustering and PCA results were interpreted as exploratory.

**Figure 8:**
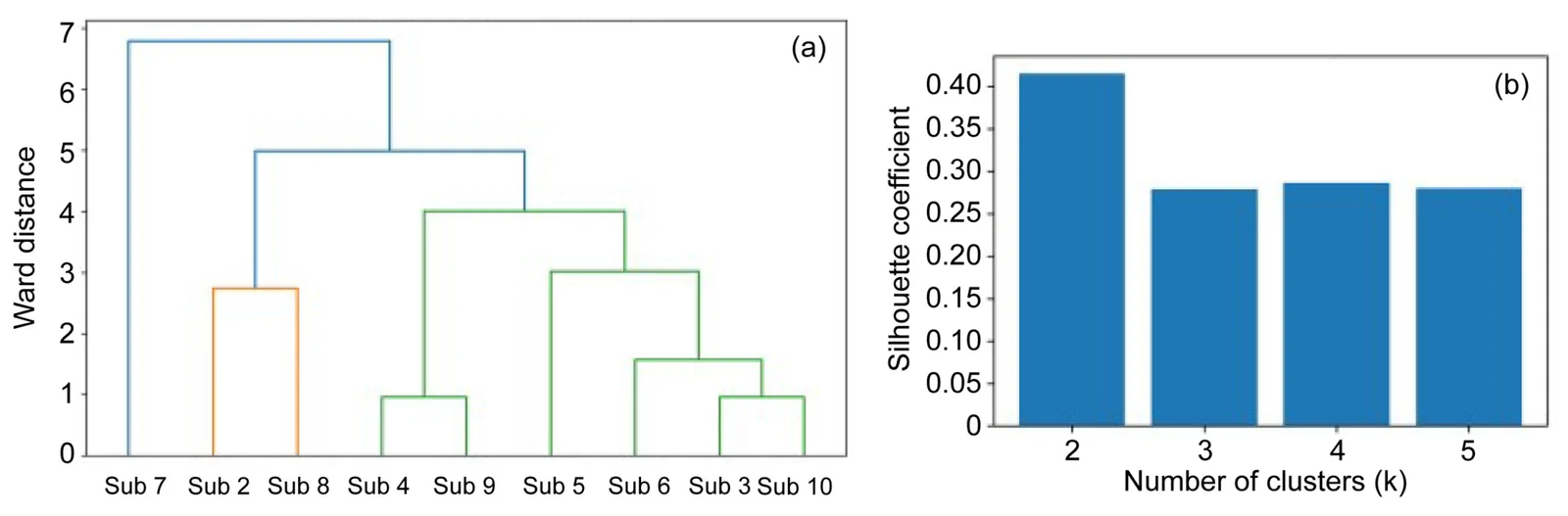
Hierarchical clustering and silhouette analysis. (**a**) Ward dendrogram based on standardized circadian–behavioral coupling features after excluding Sub 1. Branch colors indicate the three clusters in the exploratory k = 3 solution. (**b**) Silhouette coefficients for k = 2–5. The highest coefficient was observed at k = 2, whereas k = 3 was used as an exploratory interpretation-oriented solution.

Hierarchical clustering was performed using the standardized coupling features after excluding Sub 1. The Ward dendrogram and silhouette analysis are shown in Figure 8. The silhouette coefficient was highest at k = 2, indicating that the two-cluster solution provided the most parsimonious representation of this dataset. In this solution, Sub 7 was separated from the remaining participants, primarily because of its large day-to-day variability in both *t_BP_* and *t_sleep,mid_*. The k = 2 and exploratory k = 3 cluster assignments are summarized in Table 2, and the complete feature set is provided in Table S1. Because no external ground truth labels were available, confusion matrices were not applicable, and the cluster labels were used only to visualize relative participant grouping.

The exploratory three-pattern interpretation was used to further describe the heterogeneity observed in the dendrogram and feature space. Sub 7 represented a high-variability coupled pattern, with large variability but a high correlation between the two markers. Sub 2 and Sub 8 represented a low-correlation or weak-coupling pattern, whereas the remaining participants formed a main coupled pattern with relatively stable phase relationships. This interpretation is hypothesis-generating and requires validation in larger, more diverse, and independent cohorts.

**Figure 9:**
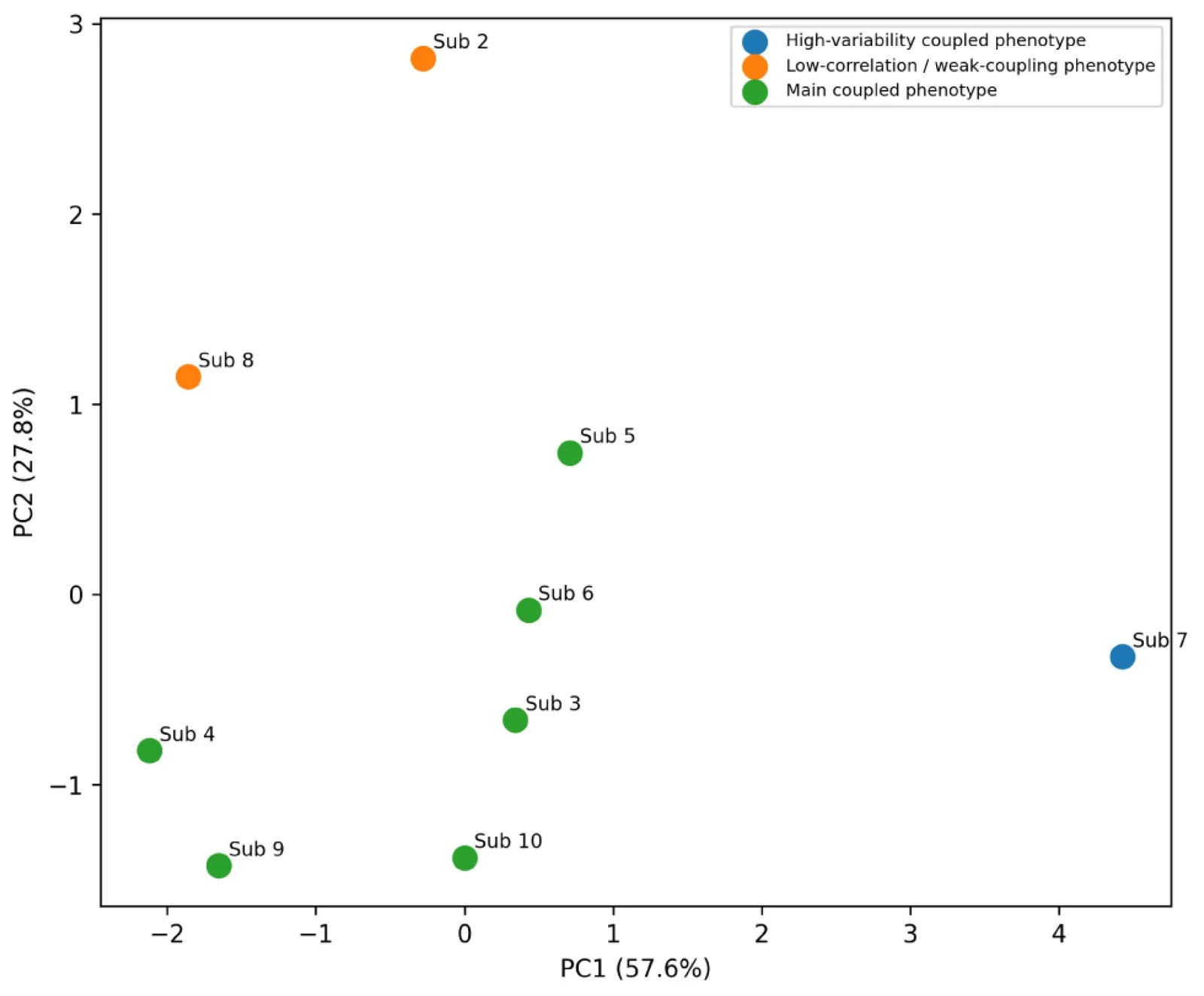
PCA based only on circadian–behavioral coupling features. PC1 mainly represents overall day-to-day variability in *t_sleep,mid_* and *t_BP_* timing, whereas PC2 represents the degree of phase-angle instability and reduced sleep-CBT correlation. The spatial arrangement was broadly consistent with the exploratory three-pattern interpretation.

Figure 9 presents the PCA based on circadian–behavioral coupling features, with the corresponding loadings provided in Table S2. PC1 had positive loadings for the circular standard deviations and day-to-day changes of both *t_BP_* and *t_sleep,mid_*, indicating that it primarily represented overall rhythm–timing variability. PC2 had a positive loading for phase-angle variability and a negative loading for the correlation between *t_BP_* and *t_sleep,mid_*, indicating that it represented coupling instability or decoupling. The PCA was used to visualize participant positions in coupling feature space. Confidence ellipses were not included because the small number of participants in each exploratory group would make them statistically unstable and potentially misleading.

**Figure 10:**
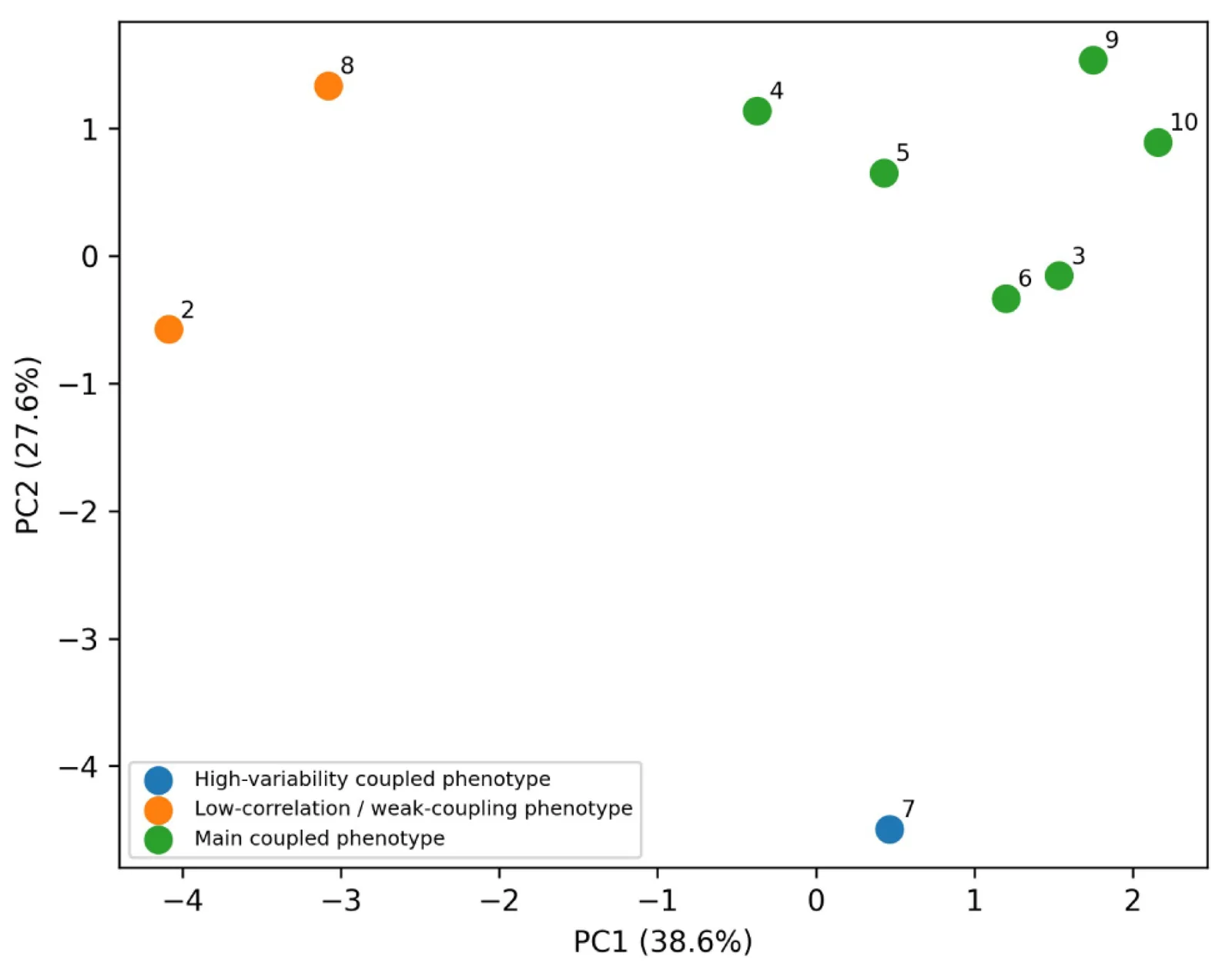
Integrated PCA using circadian–behavioral coupling, melanopic EDI, activity, and lifelog-derived behavioral features. The PCA was performed using standardized features derived from circadian–behavioral coupling, melanopic EDI, PIM-based activity, and 30 min lifelog data. Each point represents one participant. PC1 mainly separated participants with preserved coupling from those showing coupling instability accompanied by differences in light exposure, activity timing, and behavioral timing. PC2 reflected the contrast between sleep/CBT/behavioral timing variability and sleep-time low-light conditions. This analysis was used for exploratory visualization and not for confirmatory cluster validation.

Figure 10 presents the integrated PCA combining circadian–behavioral coupling features with melanopic EDI, activity, and lifelog-derived behavioral features. Definitions of the additional variables are provided in Table S3, and the PCA loadings are provided in Table S4. The largest absolute loadings on PC1 were observed for the correlation between *t_BP_* and *t_sleep,mid_*, mean melanopic EDI during the 3 h before sleep onset, variability in the activity midpoint, duration of daily exposure to melanopic EDI ≥ 250 lx, and phase-angle variability. PC1 therefore represented preserved coupling versus coupling instability, accompanied by differences in light exposure, activity timing, and behavioral timing. PC2 was primarily associated with the percentage of sleep time under melanopic EDI ≤ 10 lx, the circular standard deviations of *t_BP_* and *t_sleep,mid_*, and the timing variability of major daily behaviors, indicating a contrast between rhythm–timing variability and the sleep-time light environment.

Participants’ positions in the integrated PCA were broadly similar to those obtained from the coupling feature PCA. This similarity suggests that the coupling features captured the dominant participant-level structure, whereas the added light, activity, and lifelog variables provided contextual information for interpreting that structure. These additional features were therefore interpreted descriptively rather than as evidence of causal environmental or behavioral regulation.

## 5. Discussion

This study demonstrated the feasibility of a multimodal wearable and lifelog monitoring workflow for assessing circadian–behavioral coupling under free-living conditions. The workflow integrated a heat-flux-based wearable temperature-estimation sensor, a wrist-worn actigraph, melanopic EDI measurement, and 30 min web-based lifelog entries on a common time axis. Its principal contribution lies in wearable sensor integration and multi-sensor data fusion for daily life rhythm assessment, rather than in the development or validation of new sensor hardware. Because the cohort was small and homogeneous, the findings should be regarded as exploratory rather than population-representative.

The mean absolute difference between wearable-derived *t_BP_* and gastrointestinal-temperature-derived *t_BP_* was 27.1 ± 16.6 min. Gastrointestinal temperature measured using an ingestible capsule is a practical comparator under free-living conditions, but is not an absolute gold-standard measure of true core body temperature. Capsule location, gastrointestinal transit, food and fluid intake, local intestinal conditions, and temporal response may all affect the recorded values. Accordingly, the observed difference represents practical agreement between two temperature-derived rhythm markers rather than the absolute error of the wearable sensor. Although the 6 h post-ingestion cutoff was applied to reduce acute ingestion-related disturbances, it could not eliminate all variation associated with capsule transit or location. Nevertheless, the wearable sensor offers the practical advantage of noninvasive, continuous monitoring over several days.

In this study, *t_BP_* was used as the circadian-phase-related temperature rhythm marker, whereas midsleep time was used as a behavioral timing marker. Their temporal difference was defined as the phase angle, and coupling stability was evaluated using phase-angle variability and the correlation between the two markers. This approach extends conventional actigraphy-based rhythm assessment by evaluating not only the regularity of sleep timing, but also its temporal relationship with a physiological rhythm marker. Individuals with similar sleep timing variability may differ in their degree of alignment with the temperature rhythm; therefore, combining physiological and behavioral timing markers may provide information that cannot be obtained from activity monitoring alone.

Sub 1 was excluded from the main clustering analysis because the measurement period included overseas travel and was considered a special case reflecting re-entrainment after time zone exposure. Among Sub 2–Sub 10, silhouette analysis indicated that (k = 2) was the most parsimonious clustering solution, in which Sub 7 was separated from the remaining participants because of its large day-to-day variability in both *t_BP_* and *t_sleep,mid_*. The exploratory (k = 3) solution was used only to aid the interpretation of heterogeneity within this small dataset. Sub 7 showed a highly variable but coupled pattern, Sub 2 and Sub 8 showed low-correlation or weak-coupling patterns, and the remaining participants showed relatively stable phase relationships. These patterns should be viewed as hypothesis-generating descriptions rather than definitive phenotypes or clinical subtypes.

Because the clustering was unsupervised and no external ground truth labels were available, confusion matrices were not applicable. Confidence ellipses or statistically defined cluster boundaries were also not presented because the small number of participants in each exploratory group would make them unstable and potentially misleading. Figure 8, Figure 9 and Figure 10 were therefore interpreted as exploratory visualizations of participant-level feature space. In the PCA based on coupling features, PC1 primarily reflected overall day-to-day variability in *t_BP_* and *t_sleep,mid_*, whereas PC2 reflected coupling instability characterized by greater phase-angle variability and lower correlation between the two markers.

The integrated PCA, which additionally included melanopic EDI, PIM-based activity, and lifelog-derived features, produced a participant arrangement broadly similar to that obtained from coupling features alone. This suggests that the coupling features captured the dominant interindividual structure in this dataset, whereas light exposure, activity timing, and behavioral timing provided contextual information for interpreting that structure. The PCA loadings were interpreted descriptively, and do not establish the causal effects of environmental or behavioral factors on circadian–behavioral coupling. From a sensing perspective, however, the results support the value of integrating physiological, behavioral, environmental, and self-reported data streams. The proposed workflow should currently be regarded as an exploratory framework for visualizing participant-level relationships rather than a validated tool for clinical classification or population screening.

The workflow may be useful in future studies of personalized circadian health monitoring. Actigraphy describes sleep–activity patterns, but does not directly assess their temporal relationship with a circadian-phase-related physiological marker. Combining wearable temperature estimation, actigraphy, melanopic light exposure, and lifelog data provides a practical means of visualizing how daily behaviors relate to temperature rhythms. In larger studies, this approach may help identify unstable coupling, weak coupling, or large but synchronized shifts between sleep and temperature timing. Individualized feedback or clinical application, however, will require validation in larger, more diverse, and longitudinal cohorts.

This study has several limitations. First, the cohort consisted of only ten healthy young adults, and the main clustering and PCA analyses included nine participants after exclusion of Sub 1. The participants were similar in age, the sex distribution was unbalanced, and measurements were conducted within a limited seasonal window. These characteristics limit the generalizability of the findings, the reliability of PCA loading interpretation, and the estimation of confidence intervals for individual participant correlations.

Second, the approximately one-week monitoring period was sufficient for demonstrating feasibility and extracting preliminary participant-level rhythm features, but not for evaluating long-term reliability, seasonal stability, or reproducibility. The available ingestible capsule recording duration also varied among participants, which affected the number and timing of comparable *t_BP_* estimates. Although data obtained within the first 6 h after ingestion were excluded, residual variation related to gastrointestinal transit and local conditions remained possible.

Third, lifelog data were self-reported at 30 min intervals, and may therefore contain missing entries, recall errors, or limited precision for short-duration events. These data should be interpreted as contextual behavioral indicators rather than precise event-level measurements. In addition, the 10 min resampling and centered moving-average smoothing spanning 30 min before to 30 min after each time point were selected for rhythm-level feature extraction, and were not optimized using the present dataset. The effects of alternative smoothing windows should be examined in future studies.

Finally, the analyses focused mainly on daily and participant-level rhythm features, and did not include detailed time-resolved error modeling under different activity, posture, environmental, or behavioral states. Future studies should include larger and more diverse cohorts, longer and repeated monitoring periods, appropriate repeated-measures models, external validation datasets with comparable synchronized multimodal measurements, and state-dependent sensor error analyses to establish the robustness and broader applicability of the proposed workflow.

## 6. Conclusions

This study presented a multimodal wearable and lifelog monitoring workflow for assessing circadian–behavioral coupling under free-living conditions. The workflow synchronized wearable temperature estimates, actigraphy, melanopic EDI measurements, and web-based lifelog entries on a common time axis. The temporal midpoint between the descending and ascending mid-level crossings around the nocturnal temperature trough was used as a circadian-phase-related temperature rhythm marker, whereas midsleep time was used as a behavioral rhythm marker. Their temporal difference was defined as the phase angle, and coupling stability was evaluated using phase-angle variability and the correlation between the two markers.

The wearable-derived temperature rhythm marker showed a mean absolute difference of 27.1 ± 16.6 min from the corresponding marker derived from gastrointestinal temperature measured using an ingestible capsule. This finding indicates practical agreement between rhythm markers derived from the two measurement methods, and should not be interpreted as absolute validation against true core body temperature.

After excluding one participant who traveled overseas, clustering and PCA provided an exploratory visualization of interindividual differences in sleep–temperature rhythm coupling. Although silhouette analysis favored a two-cluster solution, the three-pattern interpretation was used only as a hypothesis-generating framework, and should not be regarded as a definitive phenotype classification. The integrated analysis further suggested that melanopic light exposure, activity timing, and behavioral timing may provide contextual information for interpreting coupling patterns in this small cohort. However, the PCA and loading-based results do not establish causal environmental or behavioral determinants.

Overall, these findings support the feasibility of integrating physiological, behavioral, environmental, and self-reported data to assess circadian–behavioral relationships in daily life. The principal contribution of this study lies in wearable sensor integration and multi-sensor data fusion, rather than in the development of new sensor hardware. Because the cohort was small and demographically and seasonally limited, larger and more diverse cohorts, longer monitoring periods, and further validations of preprocessing and sensor error characteristics are required before the workflow can be applied to population-level, longitudinal, or clinical circadian health assessment.

## Figures and Tables

**Table 1:** Comparison of *t_BP_* estimated from CALERA Research and e-Celsius. The eligible comparison window extended from 6 h after capsule ingestion until the end of the capsule recording. The ingestible-capsule recording duration was 24.8 ± 12.6 h (mean ± SD). The table contains 10 paired daily estimates from eight participants, including only daily segments for which both temperature data streams were available after preprocessing. Signed differences were calculated as CALERA Research minus e-Celsius. The summary value represents the mean absolute difference ± SD.

Subject	*t_BP_* from Calera (hh:mm)	*t_BP_* from e-Celsius (hh:mm)	Difference (min)
Sub 1	8:45	8:45	0
Sub 2	7:01	6:53	8
Sub 4 (day 1)	5:52	5:18	34
Sub 4 (day 2)	5:18	4:57	21
Sub 5	3:27	3:13	14
Sub 6	3:55	3:21	34
Sub 8	4:59	4:27	32
Sub 9 (day 1)	2:22	1:25	57
Sub 9 (day 2)	2:36	1:57	39
Sub 10	3:56	4:28	−32
Mean ± SD	-	-	27.1 ± 16.6

**Table 2:** Circadian–behavioral coupling features and cluster assignments.

Subject	Circular SD of *t_sleep,mid_*	Circular SD of *t_BP_*	SD of Phase Angle	Corr. Coeff. of *t_BP_* and *t_sleep,mid_*	Group (k = 2)	Group (k = 3)	Exploratory Interpretation (k = 3)
Sub 2	0.59	0.92	1.24	−0.30	1	1	Low-correlation/weak-coupling pattern
Sub 3	0.78	0.89	0.51	0.82	1	2	Main coupled pattern
Sub 4	0.30	0.40	0.34	0.55	1	2	Main coupled pattern
Sub 5	0.46	1.16	0.89	0.73	1	2	Main coupled pattern
Sub 6	1.02	0.69	0.73	0.70	1	2	Main coupled pattern
Sub 7	1.56	1.45	0.68	0.90	2	3	High-variability coupled pattern
Sub 8	0.56	0.30	0.71	−0.31	1	1	Low-correlation/weak-coupling pattern
Sub 9	0.53	0.33	0.31	0.85	1	2	Main coupled pattern
Sub 10	0.80	0.68	0.32	0.92	1	2	Main coupled pattern

## Data Availability

The data are not publicly available because they contain potentially identifiable information from human participants, including free-living physiological time-series data and daily activity/sleep patterns. Data sharing is restricted by ethical and privacy considerations related to the informed consent obtained from participants.

## Supplementary Materials

The following supporting information can be downloaded at: https://www.mdpi.com/article/10.3390/s26185703/s1, Figure S1: Time-series data of core body temperature, physical activity, and melanopic EDI for all participants. (a) sub 1, (b) sub 2, (c) sub 3, (d) sub 4, (e) sub 5, (f) sub 6, (g) sub 7, (h) sub 8, (i) sub 9, and (j) sub 10. Two types of core body temperature data are shown: Calera Research (red line) and e-Celsius (orange line). For core body temperature and physical activity, sleep periods are shaded in purple. For melanopic EDI, regions with values below 10 lx and above 250 lx are shaded in blue and yellow, respectively; Figure S2: Time-series data of participants’ behavioral logs. (a) sub 1, (b) sub 2, (c) sub 3, (d) sub 4, (e) sub 5, (f) sub 6, (g) sub 7, (h) sub 8, (i) sub 9, and (j) sub 10. “Out (In)” in the legend means indoor activity away from home. “Out (Out)” in the legend means outdoor activity away from home; Table S1: Full coupling features and corrected cluster assignments; Table S2: PCA loadings for coupling-feature PCA (main Figure 7); Table S3: Multimodal light, activity, and lifelog-derived features used for integrated exploratory analysis; Table S4: PCA loadings for integrated multimodal PCA (main Figure 8).
