# Supplementary material for: Integrating Wearable Temperature, Activity, Melanopic Light, and Lifelog Data to Assess Circadian–Behavioral Coupling in Free-Living Conditions
Source: Sensors (Basel). 2026 Sep 8;26(18):5703. doi: 10.3390/s26185703 (PMC13611563; doi:10.3390/s26185703)
Supplement: Supplementary file 1 [file sensors-26-05703-s001.zip › sensors-4401259-supplementary.pdf]

Supplementary Material

Integrating Wearable Temperature, Activity, Melanopic Light, and Lifelog Data to Assess Circadian–Behavioral Coupling in Free-Living Conditions

Sora Matsumoto, Yuki Hashimoto, Yoshifumi Nishida

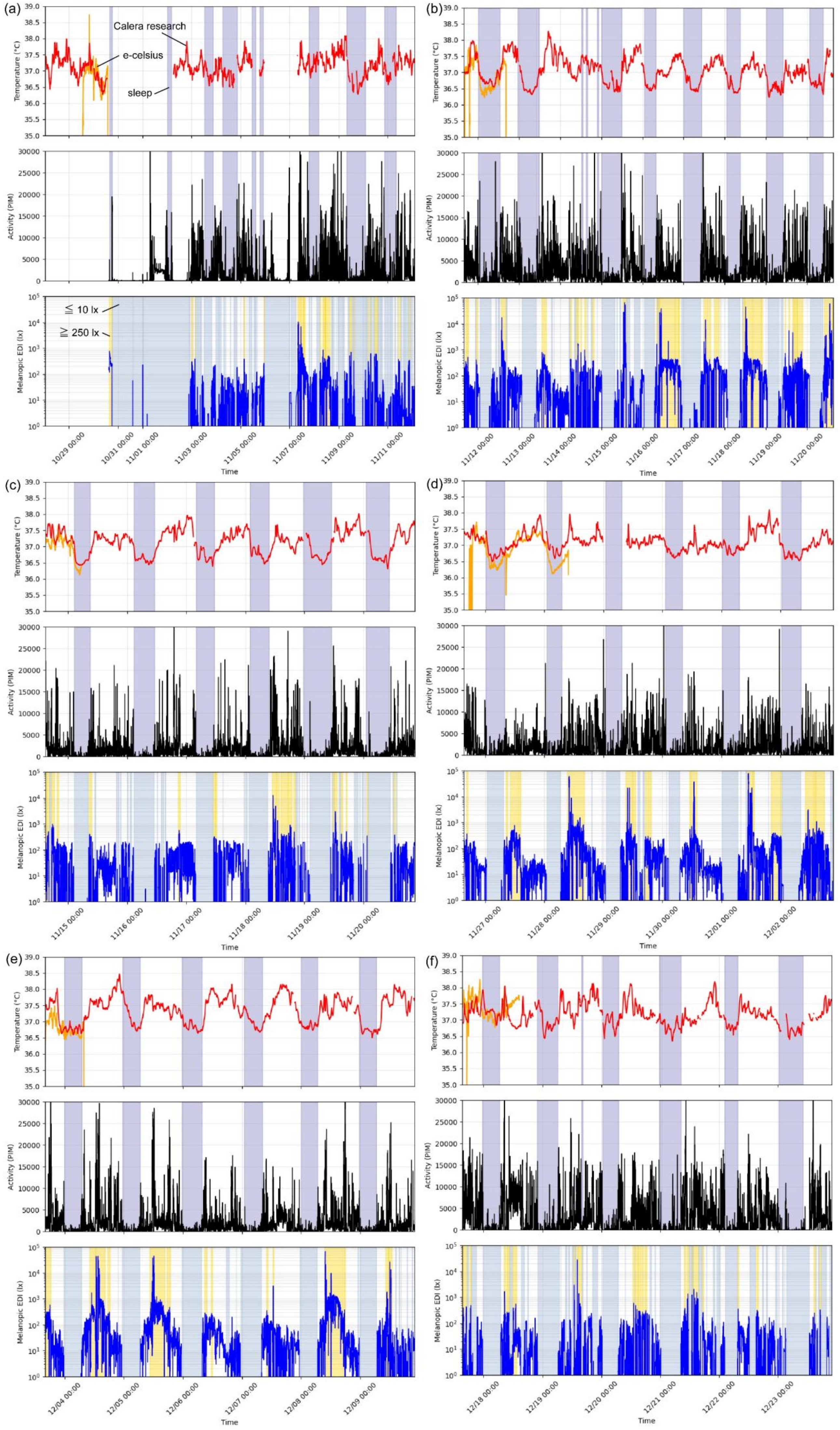

(a)
(b)
(c)
(d)
(e)
(f)
Calera research
e-celsius
sleep
≦ 10 lx
≧ 250 lx
Temperature (°C)
Activity (PIM)
Melanopic EDI (lx)
Time
10/29 00:00
10/31 00:00
11/01 00:00
11/03 00:00
11/05 00:00
11/07 00:00
11/09 00:00
11/11 00:00
11/12 00:00
11/13 00:00
11/14 00:00
11/15 00:00
11/16 00:00
11/17 00:00
11/18 00:00
11/19 00:00
11/20 00:00
11/27 00:00
11/28 00:00
11/29 00:00
11/30 00:00
12/01 00:00
12/02 00:00
12/04 00:00
12/05 00:00
12/06 00:00
12/07 00:00
12/08 00:00
12/09 00:00
12/18 00:00
12/19 00:00
12/20 00:00
12/21 00:00
12/22 00:00
12/23 00:00

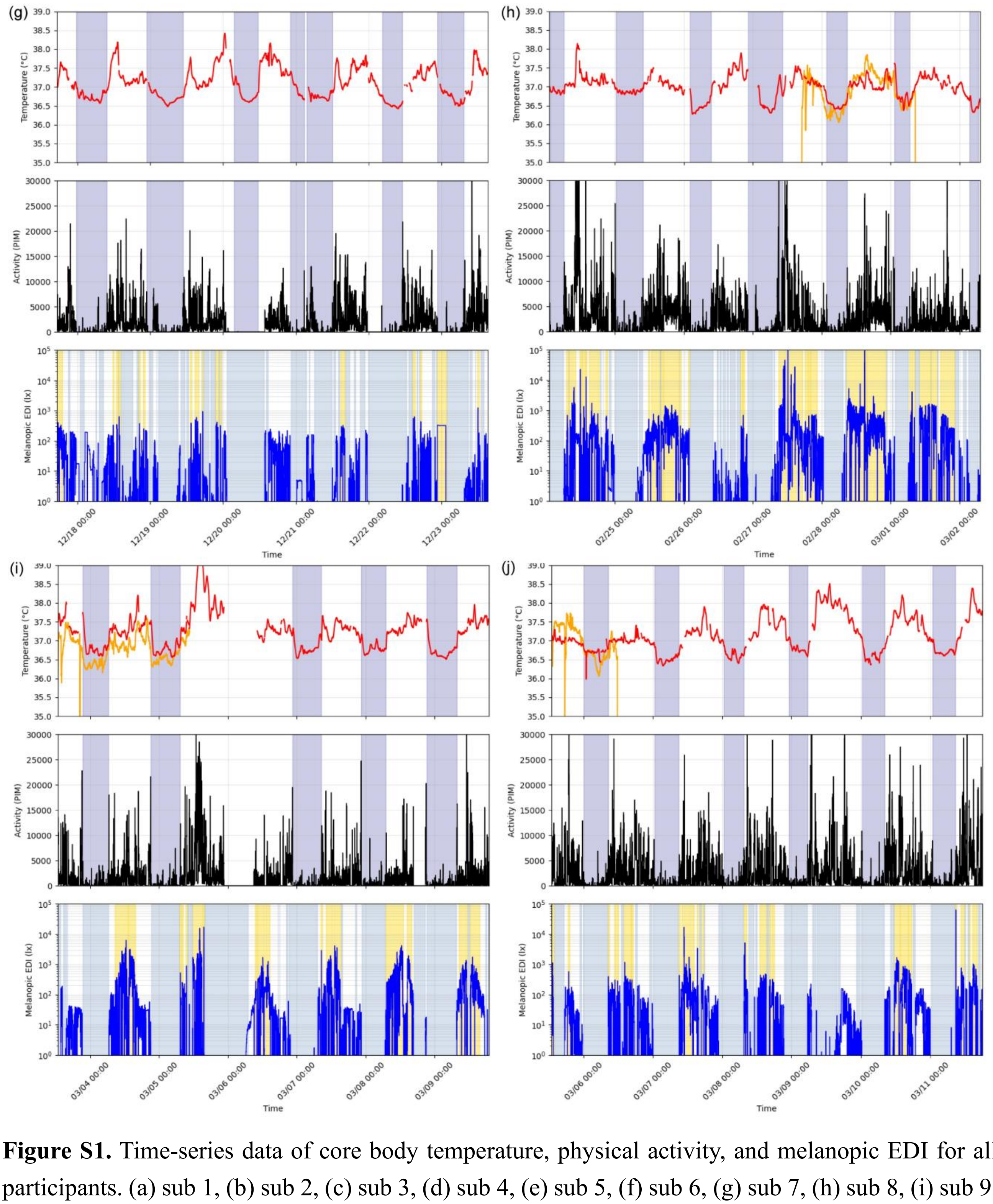

Figure S1. Time-series data of core body temperature, physical activity, and melanopic EDI for all participants. (a) sub 1, (b) sub 2, (c) sub 3, (d) sub 4, (e) sub 5, (f) sub 6, (g) sub 7, (h) sub 8, (i) sub 9, and (j) sub 10. Two types of core body temperature data are shown: Calera Research (red line) and e-Celsius (orange line). For core body temperature and physical activity, sleep periods are shaded in purple. For melanopic EDI, regions with values below 10 lx and above 250 lx are shaded in blue and yellow, respectively.

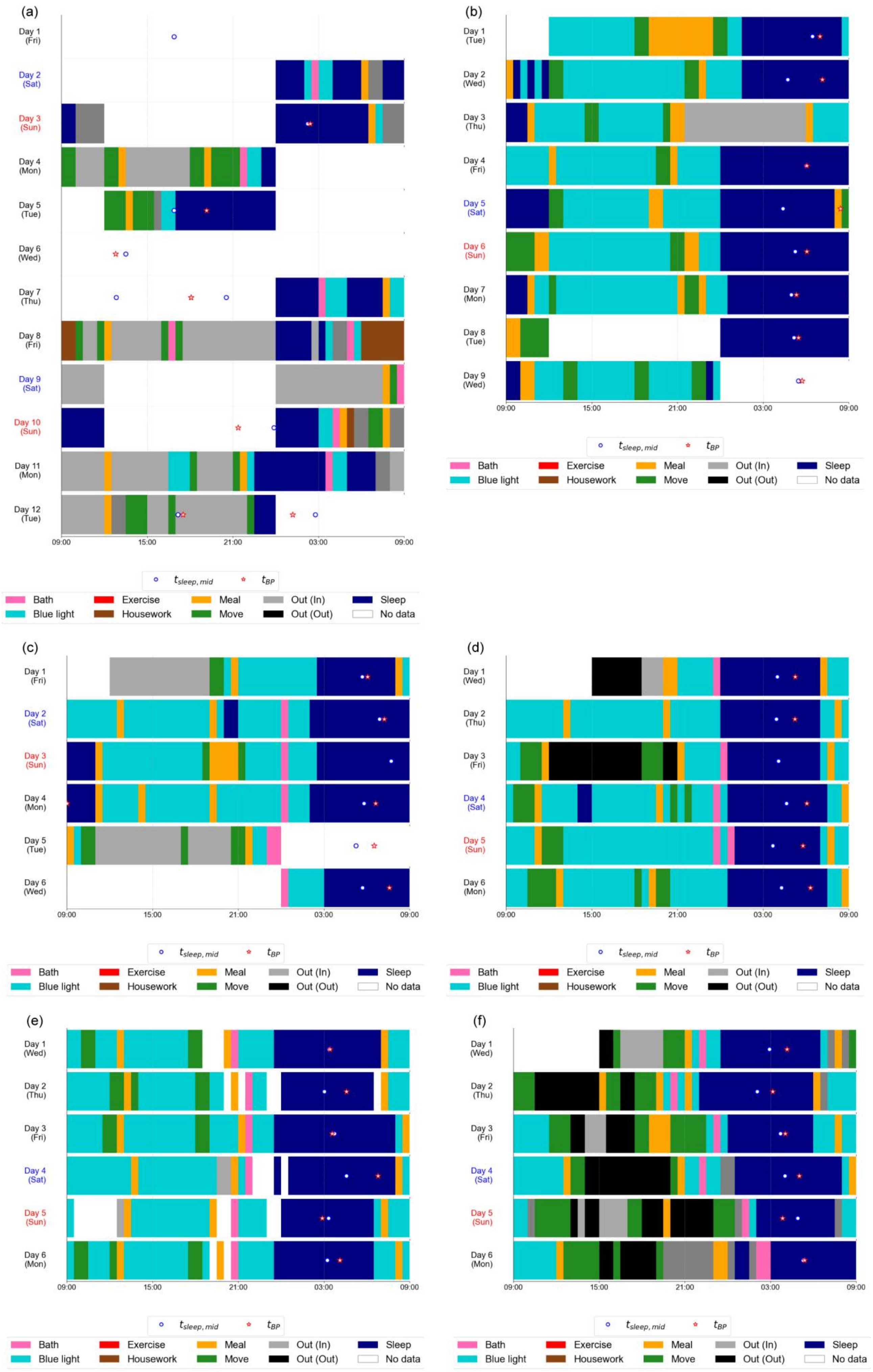

(a)
Day 1 (Fri)
Day 2 (Sat)
Day 3 (Sun)
Day 4 (Mon)
Day 5 (Tue)
Day 6 (Wed)
Day 7 (Thu)
Day 8 (Fri)
Day 9 (Sat)
Day 10 (Sun)
Day 11 (Mon)
Day 12 (Tue)
09:00
15:00
21:00
03:00
09:00
$t_{sleep,mid}$
t_{BP}
Bath
Exercise
Meal
Out (In)
Sleep
Blue light
Housework
Move
Out (Out)
No data
(b)
Day 1 (Tue)
Day 2 (Wed)
Day 3 (Thu)
Day 4 (Fri)
Day 5 (Sat)
Day 6 (Sun)
Day 7 (Mon)
Day 8 (Tue)
Day 9 (Wed)
09:00
15:00
21:00
03:00
09:00
$t_{sleep,mid}$
t_{BP}
Bath
Exercise
Meal
Out (In)
Sleep
Blue light
Housework
Move
Out (Out)
No data
(c)
Day 1 (Fri)
Day 2 (Sat)
Day 3 (Sun)
Day 4 (Mon)
Day 5 (Tue)
Day 6 (Wed)
09:00
15:00
21:00
03:00
09:00
$t_{sleep,mid}$
t_{BP}
Bath
Exercise
Meal
Out (In)
Sleep
Blue light
Housework
Move
Out (Out)
No data
(d)
Day 1 (Wed)
Day 2 (Thu)
Day 3 (Fri)
Day 4 (Sat)
Day 5 (Sun)
Day 6 (Mon)
09:00
15:00
21:00
03:00
09:00
$t_{sleep,mid}$
t_{BP}
Bath
Exercise
Meal
Out (In)
Sleep
Blue light
Housework
Move
Out (Out)
No data
(e)
Day 1 (Wed)
Day 2 (Thu)
Day 3 (Fri)
Day 4 (Sat)
Day 5 (Sun)
Day 6 (Mon)
09:00
15:00
21:00
03:00
09:00
$t_{sleep,mid}$
t_{BP}
Bath
Exercise
Meal
Out (In)
Sleep
Blue light
Housework
Move
Out (Out)
No data
(f)
Day 1 (Wed)
Day 2 (Thu)
Day 3 (Fri)
Day 4 (Sat)
Day 5 (Sun)
Day 6 (Mon)
09:00
15:00
21:00
03:00
09:00
$t_{sleep,mid}$
t_{BP}
Bath
Exercise
Meal
Out (In)
Sleep
Blue light
Housework
Move
Out (Out)
No data

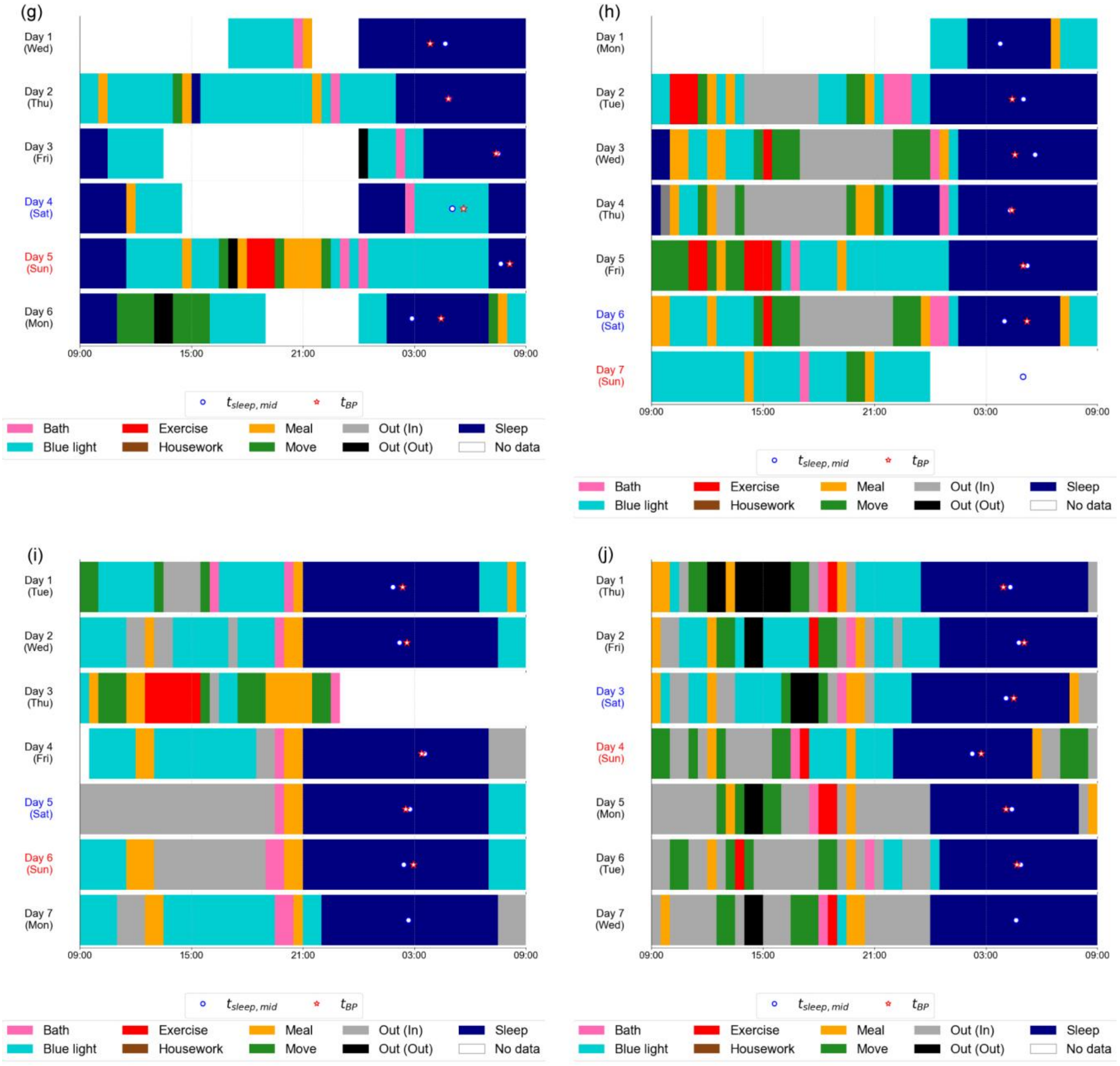

Figure S2. Time-series data of participants' behavioral logs. (a) sub 1, (b) sub 2, (c) sub 3, (d) sub 4, (e) sub 5, (f) sub 6, (g) sub 7, (h) sub 8, (i) sub 9, and (j) sub 10. “Out (In)” in the legend means indoor activity away from home. “Out (Out)” in the legend means outdoor activity away from home.

Table S1. Full coupling features and corrected cluster assignments.

Subject	Group (k=2)	Corrected interpretatio (k=2)	Group (k=3)	Corrected interpretatio n (k=3)	Circular SD of $t_{sleep,mid}$	Circular SD of t_{BP}	SD of phase angle	Diff. in $t_{sleep,mid}$ (consec. Days)	Diff in t_{BP} (consec. Days)	Corr. Coeff of t_{BP} and
Sub 2	1	All other participants	1	Low-correlation /	0.59	0.92	1.24	0.81	0.97	-0.30
Sub 3	1	All other participants	2	Main coupled	0.78	0.89	0.51	0.90	1.16	0.82
Sub 4	1	All other participants	2	Main coupled	0.30	0.40	0.34	0.50	0.44	0.55
Sub 5	1	All other participants	2	Main coupled	0.46	1.16	0.89	0.58	0.58	0.73
Sub 6	1	All other participants	2	Main coupled	1.02	0.69	0.73	0.93	0.93	0.70
Sub 7	2	High-variability	3	High-variability	1.56	1.45	0.68	2.54	2.13	0.90
Sub 8	1	All other participants	1	Low-correlation /	0.56	0.30	0.71	0.97	0.33	-0.31
Sub 9	1	All other participants	2	Main coupled	0.53	0.33	0.31	0.59	0.49	0.85
Sub 10	1	All other participants	2	Main coupled	0.80	0.68	0.32	1.04	0.97	0.92

Table S2. PCA loadings for coupling-feature PCA (main Fig. 7).

	PC1	PC2
Circular SD of $t_{sleep,mid}$	0.47	-0.11
Circular SD of t_{BP}	0.49	0.17
SD of phase angle	0.14	0.73
Diff. in $t_{sleep,mid}$ (consec. Days)	0.46	-0.04
Diff. in t_{BP} (consec. Days)	0.50	0.05
Corr. Coeff of t_{BP} and $t_{sleep,mid}$	0.22	-0.65

Table S3. Multimodal light, activity, and lifelog-derived features used for integrated exploratory analysis.

Feature	Calculation method	Interpretation
Mean melanopic EDI during the 3 h period before sleep onset	For each sleep episode, melanopic EDI was averaged from 3 h before sleep onset to sleep onset. The subject-level value was calculated as the mean across all sleep episodes.	Represents the intensity of light exposure before sleep. Higher values indicate greater pre-sleep melanopic light exposure.
Mean daily duration of melanopic EDI ≥ 250 lx	For each day, the total duration during which melanopic EDI was ≥ 250 lx was calculated in minutes. The subject-level value was calculated as the mean across days.	Represents exposure to sufficiently bright daytime melanopic light. Higher values indicate longer exposure to light levels recommended during active periods.
Mean percentage of sleep time spent under melanopic EDI ≤ 10 lx	During PIM-derived sleep periods, the percentage of valid EDI samples ≤ 10 lx was calculated for each day or sleep episode. The subject-level value was calculated as the mean across days or sleep episodes.	Represents the degree to which a dark light environment was maintained during sleep. Higher values indicate better maintenance of low-light conditions during sleep.
Day-to-day variability in the activity midpoint	For each day, the activity midpoint was calculated as the circular weighted mean of clock time, using PIM values as weights. The subject-level value was calculated as the circular SD of daily activity midpoints.	Represents the regularity of the timing of daily activity. Higher values indicate greater day-to-day variability in the timing of activity.
Mean daily total physical activity	For each day, PIM values were summed across the day. The subject-level value was calculated as the mean daily total PIM across days.	Represents the average amount of daily physical activity. Higher values indicate greater overall activity.
Mean timing variability of major daily behaviors	For each behavior category (sleep, blue light, meal, and move), the circular SD of the clock times at which the behavior was recorded was calculated. The subject-level value was calculated as the mean of these four timing SDs.	Represents the overall regularity of daily behavioral timing. Higher values indicate more irregular timing of daily behaviors.
Mean daily duration of evening blue-light-related behavior	The number of 30-min bins labeled as blue light between 18:00 and 24:00 was counted and multiplied by 30 min. The total was divided by the number of recorded days.	Represents self-reported evening blue-light-related behavior. Higher values indicate longer evening exposure to blue-light-related activities.

Table S4. PCA loadings for integrated multimodal PCA (main Fig. 8).

	PC1	PC2
phase_angle_SD_h	-0.34	-0.18
sleep_CBT_corr	0.47	-0.11
sleep_SD_circ_h	0.13	-0.51
CBT_SD_circ_h	0.04	-0.47
Mean melanopic EDI during the 3 h period before sleep onset	-0.40	-0.01
Mean daily duration of melanopic EDI ≥ 250 lx	-0.34	0.28
Mean percentage of sleep time spent under melanopic EDI ≤ 10 lx	0.14	0.52
Day-to-day variability in the activity midpoint	-0.39	-0.08
Mean daily total physical activity	-0.22	0.10
Mean timing variability of major daily behaviors	-0.31	-0.33
Mean daily duration of evening blue-light-related behavior	-0.24	0.09
